# Lasting Developmental Effects of Neonatal Fentanyl Exposure in Preweanling Rats

**DOI:** 10.1155/2012/180124

**Published:** 2011-10-19

**Authors:** Dora Catré, Maria Francelina Lopes, António Silvério Cabrita

**Affiliations:** ^1^Faculty of Medicine, University of Coimbra, 3004-504 Coimbra, Portugal; ^2^Department of Anesthesiology, São Teotónio Hospital, EPE, 3504-509 Viseu, Portugal; ^3^Pediatric Surgery Department, Pediatric Hospital, Centro Hospitalar e Universitário de Coimbra, EPE, 3000-602 Coimbra, Portugal

## Abstract

The present study aimed to determine whether neonatal treatment with fentanyl has lasting effects on stressed developing brain. Six-day-old rats were assigned to one of three groups (10 males/group): (1) fentanyl (incision+fentanyl), (2) saline (incision+0.9% saline), and (3) unoperated (unoperated sham). Pups with a plantar paw incision received repetitive subcutaneous injections of fentanyl or vehicle through postnatal days (PNDs) 6 to 8. A nonoperated sham group served as nonstressed control. Studies included assessment of development from PND 6 to PND 21 (growth indices and behavioral testing). Fentanyl administered twice daily for three days after surgical incision had no impact on early growth and development, as measured on PND 9, but showed a lasting impact on later growth, enhanced behavioral development, and lower anxiety, as measured through PNDs 10–21. While this does not completely support a benefit from such treatment, our findings may contribute to support the neonatal use of fentanyl, when indicated, even in premature newborns.

## 1. Introduction

Fentanyl, a potent *μ*-opioid agonist, is a synthetic drug that has been widely used for pain management [1, 2] and as a general anesthetic for surgical procedures in pediatrics [3–5], namely in surgical neonatal intensive care units. In a previous study, fentanyl was identified as being within the 5 medications with the highest exposure rates in a pediatric intensive care unit [2].

Fentanyl is an appropriate medication that has rarely been linked to significant adverse effects on the central nervous system (CNS) or other systems, with proper monitoring [6]. However, concerning fentanyl use in pediatric critical care population, this is one of the many medications which are not properly tested for pediatric use [2]. 

It is well documented in clinics [7] and in experimental work [8, 9] that gradual increases of standard doses of fentanyl [7], illicit fentanyl abuse [10], drug interactions [11], or individual susceptibility may lead to severe neurotoxicity and death [10]. Animal studies have also reported adverse effects. Kofke et al. [8] evaluated the neuropathological effects of fentanyl in the brain and showed that it produces limbic system brain damage in rats and that the damage occurs over a broad range of doses. In another study,   regarding fentanyl effects in rat brain ischemia, Kofke et al. [9] showed that fentanyl, in both high and low doses, can exacerbate incomplete forebrain ischemia in rats. Additionally, it is well known from the literature that large-dose opioids in rats produce hippocampal hypermetabolism, epileptiform activity, and neuropathologic lesions [12]. These doses in rats are comparable in potency to a large-dose regimen that might be used in humans [12].

The neonatal period is a time of rapid growth and development of the brain, and perturbations to the normal series of developmental events during this time can lead to adverse functional consequences that manifest later in life. Lack of data on the impact of fentanyl's repeated use during this vulnerable period of brain development raises special concern, as is well known that the developing CNS of the neonate is recognized as very sensitive to most anesthetics, in animal research studies.

Our study's primary goal was to further investigate the possibility that repeated administration of this opiate, in a window of developmental susceptibility, could have lasting impact on neurodevelopment. It was hypothesized that neonate rat pups, exposed to both postoperative and repetitive parenteral fentanyl, would show growth restriction and abnormal neurobehavioral functions. As postnatal growth and development are sensitive measures of central neurotoxicity and brain maturation, we assessed growth and development in the infant male rat after exposing a neonate rat model of postoperative pain to repeated administration of subcutaneous fentanyl during early postnatal life.

## 2. Materials and Methods

### 2.1. Ethics Statement

Animal protocols for this study were written in strict accordance with the recommendations in the European legislation and meet the standards of the National Institutes of Health, as set forth in the Guide for the Care and Use of Laboratory Animals [13]. These protocols were approved in advance by the Ethics Committee of the Faculty of Medicine of the University of Coimbra.

### 2.2. Subjects

For this experiment, the progeny (~12 per litter) of 9 multiparous Wistar rats (Charles River Laboratories, L'Arbresle Cedex, France) was used. Litters (3 per group) were assigned to the following groups: (1) fentanyl (incision + fentanyl, *n* = 10 males), (2) saline (incision + 0.9% saline control, *n* = 10 males), and (3) unoperated (unoperated sham control, *n* = 10 males). Lactating dams were maintained with their litters for 21 days, housed in polypropylene cages in a temperature-controlled environment (20–22°C) with a 12-hour light dark cycle and *ad libitum* access to water and pelleted rat chow.

### 2.3. Procedures

**Figure 1:**
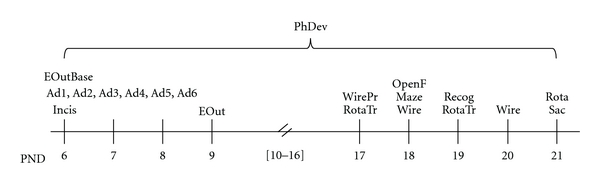
Study tests and procedures timeframe. PhDev: growth and physical development assessment (PND 6–21); EoutBase: baseline testing (righting reflex, negative geotaxis, and locomotor activity) (PND 6); EOut: early outcome assessment (PND 9); Adm: administration procedure, according to group (administration of fentanyl, administration of 0.9% saline solution, or manipulation) (PND 6–8); Incis: incision or manipulation, according to group (PND 6); WirePr: wire hanging maneuver pretest (PND 17); Wire: wire hanging maneuver test (PND 18 + 20); RotaTr: accelerating rotarod training (pretest) (PND 17 + 19); Rota: accelerating rotarod test (PND 21); OpenF: behavior in open-field arena (PND 18); Maze: elevated plus maze test (PND 18); Recog: novel object recognition test of short-term memory (PND 19); Sac: sacrifice (PND 21).

Figure 1 summarizes study tests and procedures timeframe.

Each litter was transferred together with the dam to a clean cage with fresh bedding on postnatal day 6 (PND 6). After baseline testing and weighing, rat pups received subcutaneously in the neck either the first dose of fentanyl (B∣Braun Medical Lda. Barcarena, Portugal), 25 *μ*g per kg body weight (0.1 mL per g weight of solution of fentanyl diluted to 0.25 *μ*g/mL in 0.9% saline solution), or 0.9% saline solution, 0.1 mL per g body weight; this was immediately followed by creation of a deep plantar paw incision, as previously described [14]. Briefly, the plantar aspect of the right hindpaw was prepared in a sterile manner with a 10% povidone-iodine solution. Using a no. 11 surgical blade we performed a midline incision from the heel to the base of the toes under local anesthetic (ethyl chloride spray). The underlying flexor muscle was elevated and incised longitudinally. The skin incision was closed with nylon sutures (6/0). Equivalent subcutaneous doses of fentanyl, or 0.9% saline, were repeatedly given 8–12 hours apart from the first dose for three consecutive days, with a total of 6 doses (average total dose per animal: 135 *μ*g/kg weight).

Any incidence of adverse effects (namely, respiratory depression) was recorded. Control sham animals underwent local anesthesia with skin preparation, but no incision.

On PND 9, at least eight hours after the last injection, assessment of growth and development was performed. Growth and physical development, such as teething, number of eyes open, was monitored daily through PND 6–21. The number of eyes open was scored; the observations for each item were coded as 0 (both eyes closed), 1 (1 eye open), and 2 (both eyes open). 

Rats were assessed for motor and cognitive performance between postnatal days 18 and 21 through behavioral studies in the open-field, elevated plus maze, wire hanging maneuver, novel object recognition test of short-term memory and accelerating rotarod. Between test phases and animals, apparatus, and objects were thoroughly cleansed with 70% ethanol. Behavior experiments were recorded using a camera mounted above the testing apparatus and data were reviewed without knowledge of each rats' group.

Although every litter contained pups of both sexes, growth and behavioral results reported here include data collected only from males (10 per group).

Anesthetized rats were killed on PND 21.

### 2.4. Early Outcome Assessment (Acute Effects of Fentanyl Exposure) 

#### 2.4.1. Righting Reflex

This test took place on PND 6 (baseline information) and PND 9. It consists in the time in seconds required for a pup placed on its back to right itself (all 4 paws flat on the surface). The amount of time required for the pup to right itself on all 4 limbs was measured using a stopwatch, with a maximum of 30 seconds. This test, performed as previously described, assesses subcortical maturation [15].

#### 2.4.2. Negative Geotaxis

This test took place on PND 6 (baseline information) and PND 9. It consists on the time in seconds required for a pup placed head down on a 25° incline to turn 180° and begin crawling up the slope. The cutoff time was 60 sec. The time spent for a turn of 180° upward was recorded using a stopwatch. If unsuccessful, each pup was given up to 3 trials. Each failed trial was recorded as value 61. Negative geotaxis, performed as previously described, is believed to test reflex development, motor skills and vestibular labyrinth, and cerebellar integration [15].

#### 2.4.3. Locomotor Activity

This test took place on PND 6 (baseline information) and on PND 9, at least eight hours after the last fentanyl injection. Rat pups were individually placed in a circular hole (6 cm diameter/2 cm height), and locomotor activity was scored during 3 min as follows: 1: immobility and head down; 2: raises head up; 3: forepaws over borders; 4: climbs the borders. 

### 2.5. Later Outcomes Assessment

#### 2.5.1. Behavior in the Open-Field Arena

This test took place on PND 18 and was used with the aim of assessing locomotor activity. The test was performed as previously described [16], with slight modifications. Briefly, rats were placed individually in a transparent box (60 × 40 × 25 cm) with the floor divided into twelve identical areas in a dim room. Line crossings (with all four paws placed into an adjacent area) were recorded in a 5 min period.

In addition, the presence/absence of exploratory behaviors such as rearing (standing on hind legs), grooming (using paws or tongue to clean/scratch body), and corner-facing (standing or sitting with the face directed toward the corner of the box) was recorded.

#### 2.5.2. Anxiety-Like Behavior

This test was performed on PND 18 to assess anxiety-like behavior as previously described [17], with slight modifications. Rats were placed in an elevated plus maze which consisted of a cross-shaped platform (height: 49.5 cm) with four arms (width: 10 cm; length: 110.5 cm), two of which were enclosed by walls 30.5 cm high. Each rat was placed into the central area facing an open arm and allowed to explore for 5 min. The percentage of time spent on the open arms and number of entries into the open arms were used as measures of anxiety-like behavior.

#### 2.5.3. Wire Hanging Maneuver

Wire hanging maneuver assesses neuromuscular and locomotor development [16]. This test was conducted over 3 days (maneuvers performed on PNDs 18 and 20). Rats were allowed 1 pretest on PND 17. Normal pups suspended by the forelimbs from a horizontal wire supported between two platforms (15 cm above the table top) tend to support themselves with their hind limbs, preventing falling and aiding in progression along the wire to reach the platform [16]. A sponge at the base of the apparatus served as protection for the falling rats. Latency to reach one of the platforms from the wire was measured and recorded in seconds, with a cutoff time of 60 sec. Each unsuccessful trial was recorded as value 61, with a maximum of 3 trials allowed each day. Therefore, latency values may vary from 1 to 183 per test.

#### 2.5.4. Novel Object Recognition Test of Short-Term Memory

This test, based on the natural tendency of rodents to investigate a novel object instead of a familiar one, was carried out as previously described [18]. On PND 19 each rat was allowed to move freely in an open-field box for 3 min, as habituation, followed by an exposition trial in which the rat was placed in the center of the box containing two identical objects (transparent white blocks) located in two adjacent corners. The cumulative time spent exploring each object was recorded during a 5 min period. Exploration was defined as actively touching or directly facing the object. One hour later the rats were tested for memory using the same procedure, except that one of the familiar objects was replaced with a novel different looking object. The time of exploration of each object (*t*_*n*_ and *t*_*f*_ for novel and familiar objects, resp.) was recorded for determination of the recognition index (RI): RI = *t*_*n*_/(*t*_*n*_ + *t*_*f*_).

#### 2.5.5. Rotarod

This test was performed as previously described [15], with slight modifications, to examine potential effects of fentanyl exposure on motor balance and coordination, using the accelerating rotarod (Rota Rod LI 8200; Letica SA Scientific Instruments). The rotarod test was conducted over 3 days and consisted of 2 pretests which took place on PND 17 and 19 and a test performed on PND 21. In the 2 pretests, the rats underwent habituation and training, by placement on the still rod to acclimate, followed by training on the moving rotarod, beginning at a constant speed of 5–10–20 revolutions per minute (rpm) on a schedule of three 5-minute trials. This training was repeated under the same protocol on PND 19.

On PND 21 the rats were tested using the accelerating rotarod: the apparatus was set to accelerate linearly from 4 to 40 rpm over 300 seconds. The sessions consisted of three 5-minute trials. The latency to fall from the rotarod during a 300 sec trial was recorded. Each animal was given 3 trials, and the best latency of three trials was calculated for each animal.

### 2.6. Statistics

Statistical analysis was performed using SPSS statistical software package (version 17, SPSS, Inc., Chicago, Il). Normality of distribution was determined using Kolmogorov-Smirnov and Shapiro-Wilk tests. Comparisons were made using multigroup, one-way ANOVA to test for the significance of changes among the different groups, followed by the Least Significant Difference test to compare differences between groups. If the data were not normally distributed, the Kruskal-Wallis test (nonparametric ANOVA) was used, and, where differences were identified, pairwise comparisons were performed using Mann-Whitney *U* test with appropriate correction by Holm-Bonferroni method. All differences were considered significant at *P* < 0.05. Values are expressed as means ± standard deviation (±SD) or as median and interquartile range (IQR): 25th and 75th percentiles, mean and 95% confidence interval for the mean (95% Cl), or number (percentage), as appropriate.

## 3. Results

### 3.1. Early Outcomes

There were no deaths. All animals reached PND 9.  Table 1 summarizes the most relevant outcomes.

Baseline characteristics of the groups were equivalent. No significant (*P* > 0.05) intergroup differences were found in the baseline (PND 6) body weight, locomotor activity score, and righting reflex latency. However, rats in the fentanyl group showed significantly longer baseline geotactic responses than saline (*P* = 0.005) and unoperated rats (*P* = 0.007). 

During the early period of the experiment, from PND 6 to PND 9, all rats increased body weight in a steady manner, showing no delay in physical development.

When compared to baseline results, outcomes of the fentanyl, saline and unoperated groups, recorded on PND 9, showed an improvement in all parameters, such as weight gain, enhancement of locomotor activity score, and reduced postural reflex latencies.

Comparison of the PND 9 results between fentanyl group and the controls did not show significant differences (*P* > 0.05) on righting reflex latency, negative geotactic latency, or locomotor activity score (Table 1). 

### 3.2. Later Outcomes

Table 2 summarizes the most relevant outcomes.

#### 3.2.1. Developmental and Growth Indices

As expected, all animals reached the defined endpoint (PND 21).

**Figure 2:**
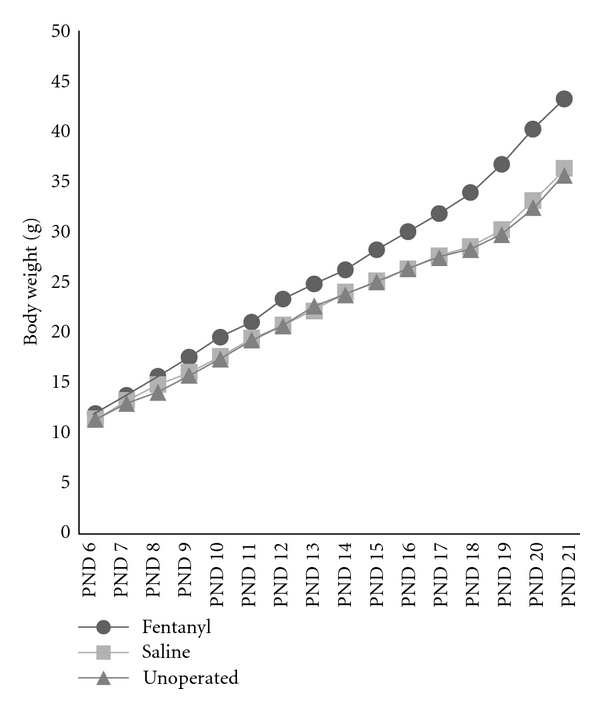
Growth curves representing mean body weight (g) through postnatal days (PNDs) 6–21. Fentanyl group showed enhanced weight gain compared to controls, after PND 12.

All rats maintained weight gain throughout the study; however, fentanyl-treated rats weighed significantly more than saline-treated and unoperated controls, from PND 12 to PND 21 (Figure 2). The mean weights (±SD) for fentanyl, saline, and unoperated rats on PND 21 were 43 (±3) g, 36 (±6) g, and 36 (±4) g, respectively. Significant differences between fentanyl versus saline (*P* = 0.001) and fentanyl versus unoperated rats (*P* < 0.001) were seen. There was no significant difference between the control groups (*P* > 0.05).

**Figure 3:**
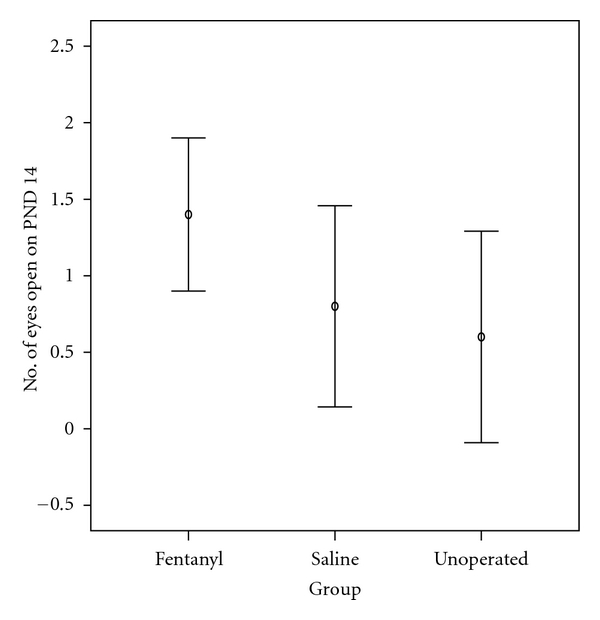
Number of eyes open on postnatal day (PND) 14. The number of eyes open was higher in the fentanyl group than in both the saline and unoperated groups (*P* > 0.05). Values represent mean and 95% confidence interval for mean.

Eyes started to open from PND 14. The median number of eyes open (scored as 0 or 1 or 2 eyes open) on this day was higher in fentanyl (2) than in saline (1) and unoperated groups (0), although without statistical significance (*P* > 0.05) (Table 2 and Figure 3).

### 3.3. Behavior in the Open Field

There were no observable intergroup differences on locomotor activity and exploratory profile. Although there was a trend for an increased mean number of line crossings in the fentanyl group (mean number: 68), compared to the saline (mean number: 62) or the unoperated ones (mean number: 48), the differences between groups were not statistically significant (*P* > 0.05), as seen in Table 2. Additionally, all rats showed a similar exploratory behavior profile, characterized by immediate beginning of locomotor activity (latency up to 20 sec) following the placement of the animal in the central area of the open field apparatus; they also showed similar profiles in the type of exploratory activity periods (with rearing and grooming behaviors) and preference for the corners exploration over that of the central area.

#### 3.3.1. Behavior during the Wire Hanging Maneuver

On PND 17 (habituation trial) all rats failed the task of platform reaching. 

**Figure 4:**
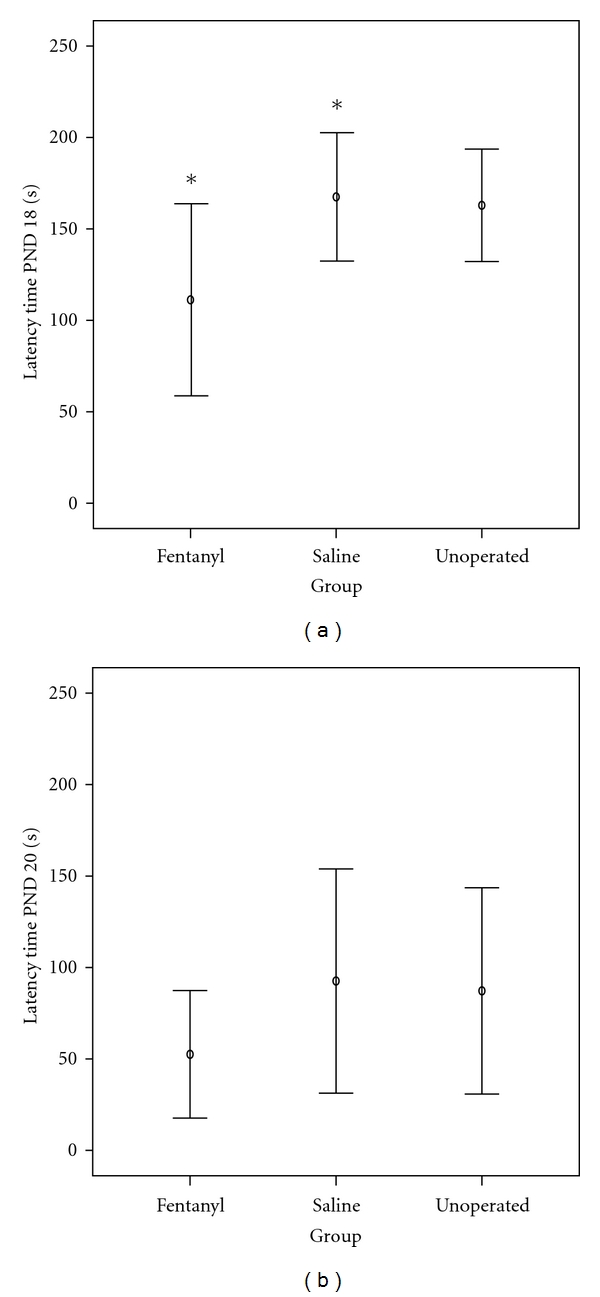
Latency times to complete the task of reaching the platform in the wire hanging maneuver on (a) postnatal day 18 and (b) postnatal day 20. Both on PND 18 and 20, rats in the fentanyl group were faster than controls, with statistical difference to the saline group (*P = 0.035) on PND 18. Values represent mean and 95% confidence interval for mean.

The success in reaching the platform differed among groups both in PND 18 and PND 20. On PND 18, 60% of the rats in the fentanyl group, 10% in the saline group, and 20% in the unoperated sham group successfully completed the task of reaching the platform; these differences were statistically significant (data not shown). Moreover, rats in the fentanyl group were faster than controls. The median latency time to complete the task in fentanyl group (127 sec) was not significantly different from that in the unoperated sham group (183 sec); however we found a significantly (*P* = 0.035) shorter median latency time in the fentanyl group (127 sec) when compared to the saline group (183 sec), as seen in Table 2 and Figure 4. 

On PND 20, while 100% of the rats in the fentanyl group completed the task successfully, only 60% in the saline and 70% in the unoperated sham groups were successful (data not shown). Again, median latency time to reach the platform was shorter (*P* > 0.05) in the fentanyl rats (25 sec) compared to saline (75 sec) or unoperated ones (76 sec), as seen in Table 2 and Figure 4. 

### 3.4. Effects of Neonatal Fentanyl Exposure on Novel Object Recognition Task of Short-Term Memory

Cognitive performance in an object recognition task of short-term memory, performed on PND 19, evidenced no adverse lasting impact in preweanling rats after neonatal exposure to six doses of fentanyl (average total dose per animal: 135 *μ*g/kg weight).

**Figure 5:**
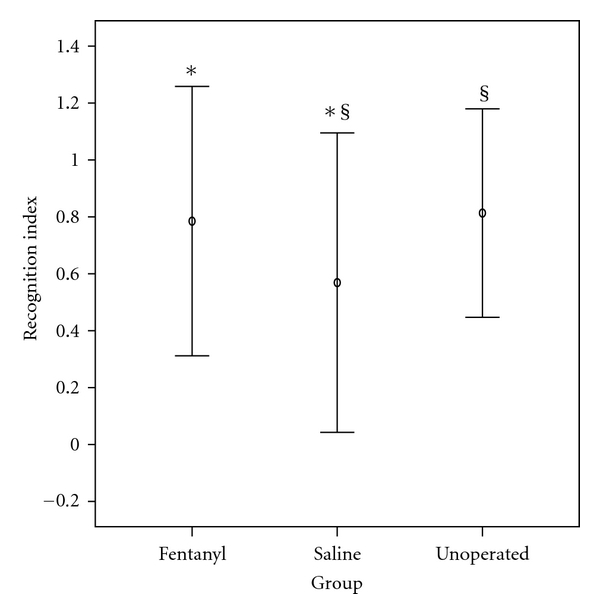
Object recognition test of short-term memory. Recognition index of novel object exploration is exploration time on new object/total exploration time (RI = *t*_*n*_/(*t*_*n*_ + *t*_*f*_)). The recognition index (RI) of a novel object in fentanyl group was not significantly different from that for the unoperated sham controls. However, a significant difference (*P = 0.045) was evidenced between the fentanyl group compared to the saline group, showing better cognitive function for the first group. A difference was also seen between the controls: unoperated sham rats displayed significantly (^§^ *P* = 0.025) better short-term memory compared to saline-treated rats. Values are mean ± SD.

The mean recognition index (RI) of a novel object in fentanyl group (0.79) was not significantly different (*P* > 0.05) from that for the unoperated sham controls (0.8). However, a significant difference (*P* = 0.045) was found between the fentanyl group (0.8) compared to the saline group (0.6), showing better cognitive function for the first group. A significant difference was also seen between the controls; unoperated sham rats displayed significant (*P* = 0.025) better short-term memory (0.8) compared to saline-treated rats (0.6), as seen in Table 2 and Figure 5.

### 3.5. Effects of Neonatal Fentanyl Exposure on Anxiety-Like Behavior

**Figure 6:**
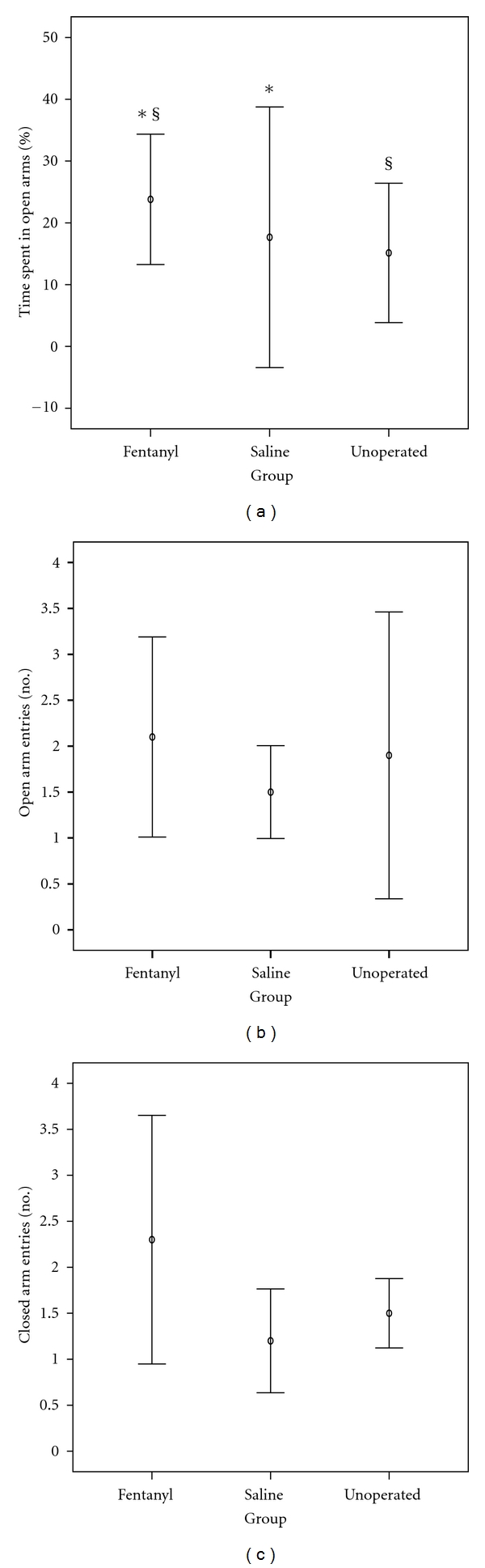
Performance in the elevated plus maze: (a) percent time in the open arms was significantly increased in fentanyl rats compared to saline rats (*P = 0.034) or unoperated controls (^§^ *P* = 0.045), suggesting that neonatal fentanyl exposure reduces some measures of anxiety-like behavior on the elevated plus maze; (b) and (c) represent the number of open arm and the number of closed arm entries, respectively, showing no significant differences between groups. Values represent mean and 95% confidence interval for mean.

Fentanyl-treated rats were significantly less anxious than the saline-treated rats (*P* = 0.035) or the unoperated ones (*P* = 0.043) in the elevated plus maze, as indicated by the increase in the median percent time spent in the open arms by the fentanyl-treated rats (18%, IQR 17–32), compared to the saline (7%, IQR 3–17) or to the unoperated sham controls (10%, IQR 7–16). However, there were no significant intergroup differences between open or closed arm entries (Table 2 and Figure 6).

### 3.6. Effects on the Accelerating Rotarod

**Figure 7:**
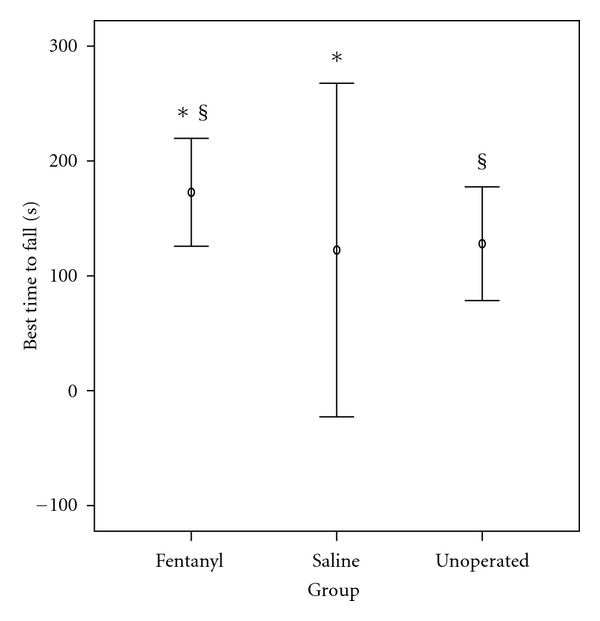
Best latency time to fall from the accelerating rotarod (speed 5–40 rpm). Rats administered fentanyl spent significantly more time on rod, compared to rats administered saline (*P = 0.022) or unoperated sham rats (^§^ *P* = 0.040). Values are mean ± SD.

The effects of fentanyl exposure on locomotor coordination and balance, as measured by the accelerating rotarod, are shown in Figure 7. 

The best (largest) of the three fall latency values (mean ± SD) achieved per rat on PND 21 was used for data analysis. Mean latency to fall from the rod for fentanyl group was 173 sec, for saline group was 123 sec, and for unoperated sham rats was 128 sec. Rats treated with fentanyl spent significantly more time on rod, compared to rats treated with saline (*P* = 0.022) or unoperated sham rats (*P* = 0.04), as seen in Table 2 and Figure 7.

## 4. Discussion

The present study was designed to assess whether exposure to repetitive injections of fentanyl during brain development influences later physical and neurological outcomes. Using a neonatal postoperative pain model, this study demonstrates, for the first time, lasting effects on growth and behavior of rat pups that underwent repeated fentanyl exposure during early postnatal life, when tested as later pre-weanling rats. The results showed that repeated fentanyl exposure of an immature stressed animal significantly interferes with growth, cognitive function, behavioral reactivity to stress, neuromuscular and locomotor development, and balance and coordination. All these outcomes (Table 2) suggest a neurological impact with possible consequences, either positive or negative, later in life. 

To examine the role of fentanyl administration in the development of behaviors that occur following repeated exposure to this medication, both in immature CNS and pain settings, we combined two strategies: the model for the study of neonatal neurodevelopment (6-day-old rat pup) was combined with the postincisional pain of Brennan paw incision [14], as a model for neonatal neurodevelopmental and postoperative pain. Translation of developmental ages from rodents to humans continues to be debated. A review paper by Vidair [19], which discusses the adequacy of the postnatal rat to serve as a model for neurodevelopment in the postnatal human, concludes that the rat in the third postnatal week is the neurodevelopmental equivalent of the newborn human and that the two species share numerous pathways of postnatal neurodevelopment. Therefore, our neonatal rat model roughly corresponds to a human premature. Brennan's model of incisional pain [14] was chosen since it simulates the usual clinical setting involving critically ill prematures in neonatal intensive care units. 

Premature newborns typically present a broad range of comorbidities which make them a complex group to study, given the many variables, painful/stressful procedures, and pharmacologic exposures involved. Therefore, experimental studies using animals allow us to exclude potential confounding variables. In our study we used a model without comorbidities in a postoperative pain and stress setting. Such a preclinical model, which leads to pain-related events that mirror the symptoms observed in patients undergoing surgery [20], gives us the opportunity to explore whether repetitive fentanyl exposure, early in neonates subject to painful stimuli, leads to later neurodevelopmental anomalies. The postoperative pain model we used was previously described by Brennan and coworkers [14]. This rat model consists of an incision of the plantar paw skin, with damage of the underlying muscle, which results in localized mechanical hypersensitivity that lasts 3–5 days. Further research by Brennan's group showed release of excitatory amino acids, such as glutamate and aspartate, activation of dorsal horn cells, and central sensitization [21].

Concerning protocol design, the dose of fentanyl used in this study, although at first sight much higher than the neonatal human recommended dose, was chosen according to the species known metabolism to relate to that typically encountered in clinical settings reflecting antinociceptive ED50 values for PND6 rats [22]. We assessed behavioral problems in our neonatal stressed model using a validated set of tests usually chosen for drug toxicity screening. 

Among major findings in the present study, we highlight the significant enhancement of weight gain in fentanyl group compared to controls, as summarized in Table 2. Neither fentanyl nor control conditions had significant effects on normal early pup weight gain. In contrast, there were significant group differences in rat weights on PND 21. Rats in the fentanyl group weighed more than those in the saline and unoperated sham groups, with the difference becoming significant around PND 12 and expanding as the pups aged until weaning. These outcomes suggest that the effects of the early postnatal exposure were subtle but, nonetheless, predisposed the pups to abnormal weight gain. Many hypotheses are possible to explain this finding, namely, metabolic derangements, behavior anomalies related to eating disorders, or decreased physical activity. An important issue that can be raised is whether the weight change is transitory or if it can continue into adulthood.

Other major findings in the present study were behavioral changes induced by administration of fentanyl in our model. Somewhat surprisingly, the results point towards an overall apparently “positive” effect on neurodevelopment, instead of the expected negative one. This “positive” impact was evidenced by an apparent lack of significant acute toxic effects on early development. Moreover, later, in infant rats who were treated with fentanyl, we found enhancement of the recognition index of a novel object, lesser anxiety-like behavior, and better performances on the wire hanging maneuver and on the accelerating rotarod. Furthermore, there was a trend for sooner eye opening in this group, suggesting that the eye command center of CNS of rats in the fentanyl group ages earlier. 

Interestingly, aversive stressful procedures performed in the current study, which should be associated with increased anxiety, seemed blunted by fentanyl treatment. In fact, fentanyl-treated rats were significantly less anxious than the saline and the unoperated rats in the elevated plus maze. This outcome is not clearly explained, but calmer subjects can probably better explain other outcomes found in this study, such as enhanced cognitive function, motor, and balance and coordination. It is possible that all these results are at least partially explained by a fentanyl impact on the development of central neuronal circuits, given the great plasticity of the CNS characteristic of the immature mammalian brain [23]. 

The effects of the impact of fentanyl on SNC are probably complex and multivariate with different possible mechanisms found in the literature, both potentially protective or detrimental, such as faster CNS myelination and enhanced neurogenesis by NeuroD activity level increase (a transcription factor essential for the development of the CNS) [24] eventually translating into enhanced performance or, on the other hand, cytotoxic lesion/blockade of the ventral hippocampus by N-methyl-D-aspartate (NMDA) receptor interference, manifesting as reduced anxiety [25]. It is well known from the literature that fentanyl modulates important cellular and molecular neuronal mechanisms, interfering not only in anatomically distributed neural network involved in generating states of anesthesia but also in mechanisms involved in hippocampus neurogenesis. In this setting, fentanyl may regulate the functions of the developing hippocampus, a region highly related to learning, memory, stress responses, and emotionality [25]. 

There is a growing body of evidence showing that drugs interfering in the SNC functions may cause pharmacologic neuroprotection or, on the opposite, detrimental effects, depending on the pathological conditions [19, 26–28]. Negative impact alerts are particularly alarming in the context of very ill preterm infants who usually present a multitude of physiological derangements and pathological pain conditions coupled with a very immature brain, therefore it is important to define safe indications and doses for the use of these drugs, such as fentanyl, in this stage. 

In conclusion, the current study is the first to demonstrate that rat pups exposed to parenteral fentanyl in a painful context have lasting growth and behavioral changes. The study highlights behavioral changes that could potentially affect brain function either in a positive or negative manner. These results should serve as a basis for further research and should lead investigators to focus on specific pathways relevant to the changes in behavior we have shown. Our findings may contribute to support the neonatal use of fentanyl, when indicated, namely in postsurgical settings, even in premature newborns. However, extrapolating our data to a clinical setting must be done with caution, as with every animal study. 

## Figures and Tables

**Table 1:** Early outcomes.

Measures	Groups			*P*
	Fentanyl	Saline	Unoperated	
Weight, mean (±SD), g:				
PND 6	12 (±1)	11 (±2)	11 (±2)	>.05
PND 9	18 (±2)	16 (±3)	16 (±2)	>.05

Righting latency, median (IQR), sec:				
PND 6	2 (1-2)	2 (2-2)	2 (2-2)	>.05
PND 9	1 (1–1.3)	1 (1-2)	1 (1–1.3)	>.05

Geotactic latency, median (IQR), sec:				
PND 6	183 (152–183)	29 (20–145)	34 (12–48)	<.05*
PND 9	19 (13–27)	23 (11–44)	22 (12–48)	>.05

Locomotor activity score, median (IQR):				
PND 6	1 (1-2)	2 (1–2.3)	1 (1-2)	>.05
PND 9	2 (1.8–3)	3 (2-3)	3 (2.8–3)	>.05

PND: postnatal day; IQR: interquartile range; *P*: significance for independent samples.

Locomotor activity scores: 1: immobility and head down; 2: raises head up; 3: forepaws over borders; 4: climbs the borders.

Fentanyl versus saline (*P* = 0.005) and fentanil versus unoperated (*P* = 0.007), both *P < .05.

**Table 2:** Later Outcomes.

Measures	Groups			*P*
	Fentanyl	Saline	Unoperated	
Weight PND 21, mean (±SD), g	43 (±3)^∗§^	36 (±6)*	36 (±4)^§^	*F versus S =.001;
				^§^F versus Un <.001;
Eye opening score, PND 14, median (IQR)	2 (1-2)	1 (0–2)	0 (0–2)	NS
Open field, line crossing, mean (±SD)	68 (±23)	62 (±24)	48 (±27)	NS
Latency time wire, median (IQR), sec:				
PND 18	127 (20–183)*	183 (183-183)*	183 (162–183)	*F versus S =0.035
PND 20	25 (15–98)	75 (12–183)	76 (9–183)	NS
Recognition index, mean (±SD)	0.8 (±0.3)*	0.6 ± (0.3)^∗§^	0.8 (±0.2)^§^	*F versus S =0.045;
				^§^Un versus S =0.025
Time (%) spent open arms, median (IQR)	18 (17–32)^∗§^	7 (3–17)*	10 (7–16)^§^	*F versus S =0.034;
				^§^F versus Un =0.045
Best latency time fall rod, mean (±SD), sec	173 (±24)^∗§^	123 (±73)*	128 (±24)^§^	*F versus S =0.022;
				^§^F versus Un =0.04

PND: postnatal day; IQR: interquartile range; *P*: significance for independent samples: NS: nonsignificant; F: Fentanyl; S: Saline; Un: Unoperated.
